# Vitamin D and hypertension: Prospective study and meta-analysis

**DOI:** 10.1371/journal.pone.0174298

**Published:** 2017-03-30

**Authors:** Dan Qi, Xiao-lu Nie, Shouling Wu, Jun Cai

**Affiliations:** 1 The Department of Cardiology, Chaoyang Hospital, Capital Medical University, Beijing, China; 2 Children’s Hospital, Capital Medical University, Beijing, China; 3 Department of Cardiology. Kailuan Hospital, North China University of Science and Technology, Tangshan, China; 4 Professor, State Key Laboratory of Cardiovascular Diseases, National Center for Cardiovascular Diseases, Fuwai Hospital, Chinese Academy of Medical Sciences and Peking Union Medical College, Beijing, China; University of Alabama at Birmingham, UNITED STATES

## Abstract

**Objectives:**

The study sought to determine the link between vitamin D concentrations and incident hypertension in prospective study and meta-analysis.

**Methods:**

The study was embedded in the Kailuan Study, a population-based cohort of adults that contains underground miners. In 2012, we studied 2,456 men and women free of prevalent hypertension, age 21 to 67 at baseline. Serum 25-hydroxyvitamin D was measured from previously frozen baseline samples using ELISA (Enzyme-Linked ImmunoadSorbent Assay). We use the logistic regression analysis to estimate the odd radio (ORs) 95% confidence intervals (CIs) for 25-hydroxyvitamin D [25(OH)D] concentrations with incident hypertension. To help place our new data in context, we conducted a systemic review and meta-analysis of previous prospective reports of vitamin D and hypertension.

**Results:**

During a median follow-up of 2 years, 42.6% of the cohort (n = 1047) developed hypertension. Compared with the 25-hydroxyvitamin D >30ng/ml, 25-hydroxyvitamin D <20 ng/ml was associated with a greater hypertension risk (OR: 1.225 [95% CI: 1.010 to 1.485] p = 0.04), although the association was attenuated and not statistically significant after adjusting for potential confounders (OR: 1.092 [95% CI: 0.866 to 1.377] p = 0.456). This meta-analysis included seven prospective studies for 53,375 participants using adjusted HR founded a significant association between vitamin D deficiencies and incident hypertension (HRs = 1.235 (95% CI: 1.083 to 1.409, p = 0.002)).

**Conclusion:**

Lower serum 25-hydroxyvitamin D concentrations were not associated with a greater risk of incident hypertension. More research is needed to further determine the role of 25-hydroxyvitamin D in hypertension prevention and therapy.

## Introduction

Resent studies have shown that vitamin D deficiency is a worldwide health problem[1–3]. The standard of 25-hydroxyvitamin D (25(OH)D) of 50 nM (20ng/ml) in blood is not achieved by 50% of the North American elderly population and by two-thirds of the rest of the world and the situation is frequently not much better in younger subjects[4]. Vitamin D has been established as a major factor influencing metabolism of bones and calcium metabolism, even diabetes mellitus, metabolic syndrome, cardiovascular disease and hypertension. There has been considerable interest in the potential relevance of vitamin D concentrations to human incident hypertension. Accumulating evidence suggests that vitamin D level is inversely related to blood pressure and risk of hypertension in observational studies in the Western populations[5–7]. Animal studies provide strong support for 1,25-dihydroxyvitamin D_3_-mediated down-regulation of renin expression and RAS (renin-angiotensin-aldosterone system) activity via its interaction with the vitamin D receptor[8]. Recent discovery that vitamin D functions as a potent negative endocrine regulator of renin gene expression provides some insights into the development of hypertension[9]. Prospective data on the relationship between vitamin D and incident hypertension are, however, controversial and little in China. Two health professional cohorts reported an inverse relation between plasma vitamin D and risk of hypertension[10,11], while other population-based studies did not find relation between plasma vitamin D and change of blood pressure or risk of future hypertension[7,12]. A community- based cohort study of 1816 Chinese participants in the Chin-Shan Community Cardiovascular Cohort Study in Taiwan suggested no association between 25(OH)D and the risk of incident hypertension[13].

Taken together, it may be hypothesized that the increased risk of hypertension in some populations may partly be explained by their poor vitamin D status. China, with a rapidly developing economy and large aging population, is experiencing a growing pandemic of hypertension. To the best of our knowledge, similar studies in Asian populations especially in China are lacking. The objective of our study is to further evaluate vitamin D inadequacy as a novel risk factor for hypertension. We examined the prospective associations of serum 25-hydrovitamin D and risk of developing hypertension in a cohort of 2,456 in China. We conducted a systemic review and meta-analysis of previous prospective reports of vitamin D and hypertension [7,10–12,14–16], as a reference, in order to better clarify the association between serum 25(OH)D concentrations and hypertension.

## Methods

### Participants

This study was embedded in the Kailuan Study[17–19], a large prospective population-based cohort study conducted among residents that contains underground miners in Kailuan, a district of Hebei, China. In 2012, there was a total of 2,456 participants were recruited, the male 81.9%, and female 18.1%. To test the stability of the estimates, we conducted several sensitivity analyses: 1) excluding participants with incident hypertension diagnosed solely by incident use of antihypertensive medications and 2) excluding participants defined as having hypertension at baseline. The study was approved by the Ethics Committees of Kailuan General Hospital, following the guidelines outlined by the Helsinki Declaration. All participants agreed to participate in the study and provided written informed consent. In our study, participants were excluded if they had any of the following characteristics during the 2012–2014 assessments.

Nurses/doctors administered questionnaires, made physical measurements, recorded the blood pressure (BP), collected nonfasting venous blood samples from which serum was stored at -20°C for subsequent analyses. All participants were followed up for all cause mortality and cardiovascular morbidity, and follow-up has been achieved for 99% of the cohort. All participants were interviewed at home and visited the research center for further examinations. Additional follow-up visits were conducted every year.

### Ascertainment of hypertension

Hypertension was identified from self-reports or doctor measurement on the baseline and follow-up measures or questionnaires meeting at least one of three JNC8 criteria: systolic blood pressure (SBP) ≥ 140 mmHg, diastolic blood pressure (DBP) ≥ 90mmHg, or use of anti-hypertensive medicines. Incident hypertension was defines as newly developed hypertension among those free of baseline hypertension. The definition of incident of hypertension is patient self-report or doctor measurement.

### Laboratory methods

We measured serum 25(OH)D concentrations in baseline samples that were collected in the morning after 8 to 12 hour overnight fast. Samples were stored at -80°C. We measured total serum 25(OH)D using the immunodiagnostic ELISA. More over, there are new vitamin D pathways recently discovered with alternative pathways of vitamin D hydroxyderivatives detectable in in human serum and tissues which included CLIA(chemiluminescent immunoassay), HPLC(High Performance Liquid Chromatography) and LC-MS(liquid chromatograph-mass spectrometer). Measured plasma 25(OH)D levels were split into 3 specified categories using the definition of vitamin D deficiency as the lowest category and ideal 25(OH)D levels as the reference category: >30 ng/mL, 20 to 29 ng/mL, and <20 ng/mL[1].

### Covariates

On the Kailuan study baseline questionnaires, people provided self-reports of age (in years), height and weight (used for calculation of body mass index [BMI] in kg/m^2^), cigarette smoking (never, past, current), alcohol use (none, current), history of diagnosed diabetes (no, yes), highest level of education (none, primary school, junior high school, high school, college or more), work environment (miners on the wall, underground miners without dust exposure, underground miners with dust exposure), sault habit (like light, normal, like salty), urine protein (-, trace, ±,+, ++, +++) and low density lipoprotein (LDL) cholesterol, triglycerides, total cholesterol, creatinine.

### Statistical analyses

All statistical analyses were conducted using SPSS 13.0 for windows 7, with a two-side significance level of 0.05. We used a Wald test to calculate p values and 95% confidence intervals (CIs). We tabulated baseline participant characteristic by widely used categories of 25(OH)D (< 20 ng/ml; > 20 to 29 ng/ml; and > 30 ng/ml). One-way ANOVA for continuous variables and χ2-tests for categorical variables were performed to test the difference in population characteristics by baseline vitamin D status. Logistic regression was employed to assess the associations between 25(OH)D and hypertension as the outcome variable. The estimates in the crude model were unadjusted. Multivariable models assessed the association after adjusted for known hypertension risk factors. In the first model, OR for the outcomes was adjusted for the following potential confounders: age. The second model was further adjusted BMI, diabetes, LDL cholesterol, triglycerides, total cholesterol, highest level of education, Work environment, smoke, drink, salt habit and physical exercise. The third model further adjusted creatinine and urine protein.

A meta-analysis of studies published before May 2015 with over 1 year’s follow-up was conducted using search, abstraction, and data synthesis methods.

#### Literature search

Two authors (Dan Qi and Xiaolu Nie) independently performed a computer-based literature search with PubMed/EMBASE/MEDLINE/Clinical trials/CNKI and the Cochrane Database of Systematic Reviews from 1957 to May 2015. We use the Medical Subject Headings (MeSH) terms, which included vitamin d: cholecalciferol, 25-Hydroxyvitamin D_2_, 25(OH)D, 25-Hydroxyvitamin D, 25-Hydroxycholecalciferol, 1,25(OH)_2_D, calcifedio* and blood pressure: hypertension, hypertensi*, blood pressure; The titles and abstracts of the selected articles were examined. Full-text articles were retrieved. We also searched the reference lists of included articles for additional studies. We also searched for gray literature using Google.

#### Inclusion and exclusion criteria

An article was appropriate for our study if originated from randomized controlled trials that reported the relationship between serum 25-hydroxyvitamin D concentrations and incident hypertension. No language restrictions were applied. We further restricted the conditions to human studies, and adult subjects aged over eighteen years. Animal test and review were not included. Studies that assess the relationship between serum 25-hydroxyvitamin d concentrations and incident hypertension only in protocol or abstract form were also not included. A minimum 1-year’s follow-up was necessary for inclusion in the review to ensure the relationship had sufficient time to produce a better effect. If the same study published multiple publications, only the most recent was included.

#### Data extraction

The data extraction was independently performed by two reviewers (Dan Qi and Xiaolu Nie) which included anther, year of publication, original country, race, sample size, numbers of case and control, mean age, study population, study design, outcome, method of intervention, years of follow-up, inclusion and exclusion criteria, method of measurement of 25(OH)D and method of diagnosis of hypertension. The outcome measured with mean with SD or MD with 95% confidence intervals (CIs). If there are discrepancies between reviewers, a joint reevaluation of the original article will be addressed.

#### Risk for bias assessment

Two reviewers independently assessed the quality of each study according to the Cochrane risk of bias: Random sequence generation, allocation concealment, blinding of participants and personnel, blinding of outcome assessment, incomplete outcome data, selective reporting and other bias. We generated funnel plots to examine possible publication bias; these were supplemented by formal statistical testing using the Egger test.

#### Data synthesis and analysis

The primary outcome measured was mean difference (MD) and 95% confidence interval (95% CI). To measure the outcome, we used the random-effects model (REM). The REM accounts for heterogeneity between studies and all analyses were reported with the use of the REM. We assessed for heterogeneity with this method which gave a qualitative value and was considered statistically significant for heterogeneity if a *P* value of less than .1 was obtained, and the I2 statistic, which gives a quantitative measurement; I2 values higher than 50% were considered a reflection of severe heterogeneity.

## Result

### Associations of 25-hydroxyvitamin D concentrations with baseline characteristics

After excluding participants who had hypertension, 2,456 participants were available for analysis. During the study period, a total of 2,456 samples from the all-unique patients were tested for vitamin D concentrations.

The baseline characteristics for the patients with and without vitamin D deficiency are listed in Table 1. Among the 2,456 participants, mean age was 43.90 ± 11.58 years and 81.9% were male. The mean systolic blood pressure at baseline was 120.70±10.34, 121.14±9.94 and 121.93±9.65 in serum 25(OH)D >30 ng/mL, 20 to 29 ng/mL, and <20 ng/mL, respectively. The diastolic blood pressure was 78.50±5.43, 78.39±5.52 and 79.06±4.99, respectively. The distribution of serum 25(OH)D was unimodal and approximately symmetric, with mean values of 26.45 ± 6.89 ng/ml. A total of 621 patients (25.3%) were in the predefined sufficient (>30ng/ml), 485 (19.7%) intermediate for vitamin D (20–30 ng/ml) and 1,350 (55.0%) patients were deficient (<20 ng/ml). At baseline, compared to participants with normal vitamin D levels, people classified as being vitamin D deficient (<20ng/ml) were related to higher prevalence of type 2 diabetes and more extreme working environment (Table 1).

**Table 1:** Baseline characteristic.

c	Serum 25(OH)D (ng/ml)			p
	<20	20–30	>30	
n	1350	485	621	/
Age,yrs	44.28±11.48	43.42±11.79	44.01±11.46	0.402
Males	1099(81.4)	393(81.0)	519(83.6)	0.143
BMI, kg/m2	24.67±3.39	24.66±3.26	24.48±3.13	0.5
SBP, mmHg	120.70±10.34	121.14±9.94	121.93±9.65	0.037
DBP, mmHg	78.50±5.43	78.39±5.52	79.06±4.99	0.057
LDL cholesterol, mg/dl	2.69±1.64	2.58±0.96	2.56±0.94	0.128
TG triglycerides, mg/dl	1.57±1.68	1.52±1.63	1.55±1.58	0.853
TC total cholesterol, mg/dl	4.83±1.03	5.02±3.88	4.79±0.99	0.104
Cr creatinine, umol/l	71.45±19.42	70.70±15.90	71.30±16.78	0.76
DM				<0.001
No	1315(97.4)	474(97.7)	607(97.7)	
Yes	35(2.6)	11(2.3)	13(2.3)	
Highest level of education				0.122
None	1(0.1)	1(0.2)	1(0.2)	
Primary school	20(1.5)	4(0.8)	15(2.4)	
Junior high school	857(63.5)	325(67.0)	418(67.3)	
High school	208(15.4)	78(16.1)	99(15.9)	
College or more	204(15.1)	59(12.2)	68(11.0)	
Work environment				0.039
Miners on the wall	776(57.5)	246(50.7)	325(52.3)	
Underground miners without dust exposure	83(6.1)	26(5.4)	33(5.3)	
Underground miners with dust exposure	403(29.9)	183(37.7)	228(36.7)	
Smoke				0.132
Never	880(65.2)	323(66.6)	390(62.8)	
Has quit	49(3.5)	17(3.5)	13(2.1)	
Not quit	421(31.2)	144(29.7)	218(35.1)	
Drink				0.537
Never/has quit	895(66.3)	320(66.0)	396(63.8)	
Not quit	455(33.7)	165(34.0)	225(36.2)	
Salt habit				0.073
Like light	180(13.3)	56(11.5)	72(11.5)	
Normal	1067(79.0)	396(81.6)	483(77.8)	
Like salty	103(7.5)	32(6.6)	66(10.5)	
Urine protein				0.782
-	1249(92.5)	453(93.4)	586(94.4)	
trace	2(0.1)	1(0.2)	1(0.2)	
±	/	/	/	
+	35(2.6)	16(3.3)	7(1.1)	
++	18(1.3)	5(1.0)	7(1.1)	
+++	9(0.7)	1(0.2)	5(0.8)	
Physical exercise				0.298
Never	317(23.5)	94(19.4)	135(21.7)	
2/week	880(65.2)	342(70.5)	420(67.6)	
≥3/week	152(11.3)	48(9.9)	66(10.6)	

Values are mean±SD or n%. 25(OH)D = 25-hydroxyvitamin D; BMI = body mass index; SBP = systolic blood pressure; DBP = diastolic blood pressure; LDL = low density lipoprotein; DM = diabetes mellitus.

### Incident hypertension

Among 2,456 participants free of hypertension, 1,074 developed incident hypertension during 2 years follow-up.

### Associations of 25(OH)D and incident hypertension

Lower serum 25(OH)D categories were associated with greater unadjusted incident hypertension rates during follow-up (OR:1.225 [95% CI:1.010 to 1.485, P = 0.04] (Table 2).

**Table 2:** The result of logistic analysis.

	N	Crude model OR (95%CI)	p	Model 1 OR(95%CI)	p	Model 2 OR(95%CI)	p	Model 3 OR(95%CI)	p
Serum 25(OH)D continuous	2456	0.995(0.990,1.000)	0.05	0.996(0.990,1.001)	0.086	0.996(0.990,1.001)	0.107	0.995(0.990,1.001)	0.100
Serum 25(OH)D cut-offs									
>30	621	Ref	/	Ref	/	Ref	/	Ref	/
20–30	485	1.201(0.944,1.010)	0.136	0.844(0.692,1.031)	0.096	0.845(0.684,1.045)	0.120	0.838(0.676,1.040)	0.108
<20	1350	1.225(1.010,1.485)	0.040	0.980(0.789,1.218)	0.858	1.080(0.859,1.358)	0.511	1.092(0.866,1.377)	0.456

Values are n or hazard ratio (95% confidence interval). Model 1 is adjusted nothing. Model 2 is adjusted for age. Model 2 is model 1 plus body mass index, work environment, smoking, drinking, diabetes mellitus, low density lipoprotein, triglycerides, and total cholesterol. Model 3 is model 2 plus creatinine and urine protein.

25(OH)D = 25-hydroxyvitamin D; Ref = reference category.

Associations of 25(OH)D with hypertension were attenuated after minimal adjustments (OR:1.080 [95% CI:0.859 to 1.358] p = 0.511); further adjustment for potential confounders, plus measures of creatinine and urine protein, also progressively attenuated these associations, which were no longer statistically significant (OR: 1.092 [95% CI: 0.866 to 1.377] p = 0.456).

### Meta-analysis and systematic review of available prospective studies

**Fig 1:**
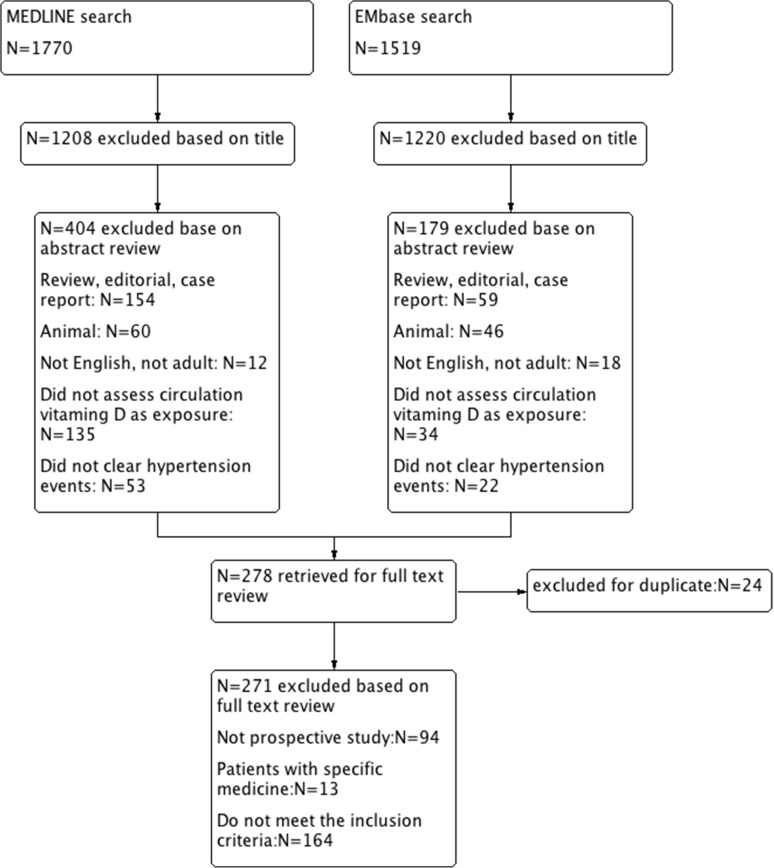
Flow diagram. Study search and flow diagram. Search terms for vitamin D included vitamin D, 25hydroxy-vitamin D, 1,25dihydroxy-vitamin D, calcidiol, and calcitriol. Search terms for hypertension included hypertension, high blood pressure, blood pressure, systolic blood pressure and diastolic blood pressure. Initial search results were further limited to English-language articles, Chinese-language articles, human studies, and studies of adults 18 years. In each box, the sum of studies in all categories may exceed the total number because of overlapping classification.

**Fig 2:**
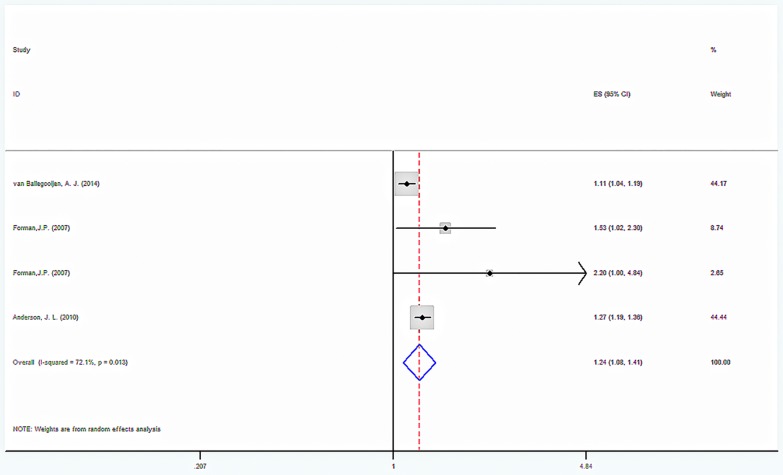
Forest plot for vitamin D sufficient VS deficient. Hazard ratios of hypertension of the deficient versus sufficient category of blood 25(OH)D concentration. CI indicates confidence interval. The size of each square is proportional to the study’s weight.

**Fig 3:**
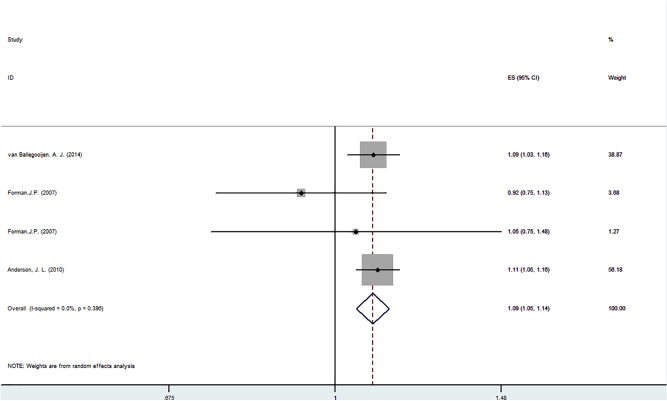
Forest plot for vitamin D sufficient VS normal. Hazard ratios of hypertension of the normal versus sufficient category of blood 25(OH)D concentration. CI indicates confidence interval. The size of each square is proportional to the study’s weight.

The results of our search strategy are summarized in Fig 1. We identified 7 relevant published prospective studies that reported on serum 25-hydroxyvitamin D concentrations and incident hypertension risk (Table 3). All of the 7 articles were published between 1989 and 2014 and involved a total 53,375 participants and reported blood pressure at baseline and follow-up. Adjusted RR/HR from the three studies were combined with meta-analysis, which found a significant reduction in blood pressure. Compared with participants with sufficient levels, those with vitamin D deficiency (<20 ng/mL) had a multivariable hazard ratio (HRs) of 1.235 (95% CI: 1.083 to 1.409, p = 0.002). There was significant heterogeneity between studies (I^2^ = 72.1% p = 0.013). The HRs was 1.093 (95% CI 1.052 to 1.136, p = 0.000) for the normal group compared with the sufficient group, but no significant heterogeneity between studies (I^2^ = 0.00% p = 0.396). The quality assessment and publication bias of recruited studies are shown in Figs 2 and 3.

**Table 3:** Baseline characteristic of meta-analysis.

Writer	Year	Number	Male	Female	HR														p for trend					
					defficency:<20ng/ml sufficency:>30ng/ml	reference	Q1:Q2			Q1:Q3			Q1:Q4			Q2:Q3					OR			
							total	LCI	UCI	total	LCI	UCI	total	LCI	UCI	total	LCI	UCI		reference	total	LCI	UCI	p
Wang, L.	2013	1211	1211	0	Q1<20ng/ml;Q4≥40ng/ml	Q1	1.02	0.77	1.36	0.74	0.55	0.99	0.91	0.68	1.2	/	/	/	0.47	/	/	/	/	/
van Ballegooijen, A. J.	2014	3002	1411	1591	Q1<20ng/ml;Q3>30ng/ml	Q3	/	/	/	1.28	1.09	1.5	/	/	/	1.23	1.06	1.41	0.003	/	/	/	/	/
Skaaby, T.	2012	4513	2279	2234	/	/	/	/	/	/	/	/	/	/	/	/	/	/	/	per 4ng/ml	1.01	0.97	1.05	0.69
Griffin, F. C.	2011	559	0	559	/	/	/	/	/	/	/	/	/	/	/	/	/	/	/	below 32ng/ml	1.1	0.61	2.1	/
Forman,J.P.	2007	1811	613	1198	Q1<15ng/ml Q3≥30ng/ml women	Q3	/	/	/	2.67	1.05	6.79	/	/	/	0.83	0.53	1.34	/	/	/	/	/	/
					Q1<15ng/ml Q3≥30ng/ml Men	Q3	/	/	/	6.13	1	37.8	/	/	/	1.12	0.51	2.48	/	/	/	/	/	/
Anderson, J. L.	2010	41497	10457	31040	Q1≤15ng/ml Q3>30ng/ml	Q3	/	/	/	1.73	1.48	2.02	/	/	/	1.26	1.12	1.42	P<0.0001	/	/	/	/	/
Forman,J.P.	2008	742	0	742	Q1≤30ng/ml Q2>30ng/ml	Q1	1.64	1.29	2.1	/	/	/	/	/	/	/	/	/	/	/	/	/	/	/

## Discussion

This was a prospective study with a selected population included underground miners with or without dust exposure, which seldom come into contact with the sun, partly avoiding sun exposure on the result of the study. In this study, we reported associations of serum 25-hydroxyvitamin D with incident hypertension in a large Chinese prospective cohort during 2 years follow-up. Lower 25(OH)D was associated with greater hypertension risk, although the association became non-significant after adjusting for potential confounders. In contrast, our meta-analysis found a significant association between 25(OH)D and hypertension. Two study was incorporated into our meta-analysis, however the data in this study could not be merged for their data is continuous variables[12]. The Inter99[12] study randomly assigned 6,784 individuals into a prospective study with 5-year follow-up. In multivariable logistic regression analyses, the odds ratios per 10 nmol/l (4 ng/ml) higher baseline vitamin D levels were 0.95 (p <0.05) and 0.94 (p = 0.01) for the development of the metabolic syndrome and hypercholesterolemia, respectively. There was no association between vitamin D and blood pressure. Moreover, Griffin *et al*. suggested that vitamin D insufficiency was associated with increased risk of systolic hypertension (OR 3.0; 95% CI (1.01, 8.7)) with 14-year follow-up in a cohort of Caucasian women after adjusting for age, body fat percentage, antihypertensive medication use, and smoking[14]. From another perspective, Wang *et al*. found after multivariable adjustment, the hazard ratios (HRs) and 95% CIs for hypertension across increasing quartiles of circulating vitamin D metabolites were 1.00 (ref), 0.94 (0.69–1.27), 0.69 (0.50–0.96), and 0.82 (0.60–1.13) for 25(OH)D (p, trend: 0.43), suggested the evidence for an inverse association between plasma 25(OH)D and risk of hypertension[10]. Women in the lowest compared with highest quartile of serum 25(OH)D had an adjusted odds ratio for incident hypertension of 1.66 (95% CI: 1.11 to 2.48; P for trend = 0.01) in Forman’s study[16]. Plasma 25(OH)D levels are inversely and independently associated with the risk of developing hypertension.

Vitamin D is very important for overall health and wellbeing. A major source of vitamin D comes from exposure to sunlight. Measurement of 25-hydroxyvitamin D in the blood and not 1,25-dihydroxyvitamin D is used to determine vitamin D status[2]. It was widely believed that vitamin D3 was solely activated through the sequential hydroxylation by CYP27A1 or CYP2R1 at C25, and by CYP27B1 at C1[20,21]. But recently studies discovered that novel metabolic pathways initiated by the CYP11A1 hydroxylases D3 can product 20-hydroxyvitamin D3[22–25]. In human epidermal keratinocytes, Slominskl *et al*. found that 20(OH)D3, 20(OH)D2 and 20,23(OH)_2_D3 inhibited cell proliferation, stimulated differentiation and inhibited NF-κB activity with potencies comparable to 1,25(OH)_2_D3 and CYP11A1 could initiate new pathways of vitamin D metabolism in a range of tissues and products[22,25]. Moreover, CYP11A1-dependent metabolism of D3 has also been detected in cells of mesenchymal origin such as dermal fibroblasts[24].

Early studies have shown that person at higher latitudes[26,27], dark skin[26,28], and winter season[29] all characterized with lower vitamin D photosynthesis from sun exposure, are associated with higher blood pressure. Subsequently, some, but not all, prospective studies demonstrated inverse association between 25(OH)D and blood pressure levels. In a subsample of the Multi-Ethnic Study of Atherosclerosis Study (MESA), comparing participants who had baseline serum 25(OH)D < 20 nmol/L versus > 30 nmol/L, the multivariable relative risk of incident hypertension was 1.28[7]. Wang *et al*.[10] found a suggestive evidence for an inverse association between serum 25(OH)D and risk of hypertension in a prospective cohort of men. Our finding is in contrast to some Chinese studies. Chan *et al*. [30]found a significant association between serum PTH and blood pressure, but not serum 25(OH)D. In Shanghai Women’s and Men’s Study, circulating 25(OH)D levels were inversely related to the levels of individual BP parameters and hypertension among middle-aged and elderly men but not among women[31]. However, Li *et al. [32]*investigated that serum vitamin D and PTH levels were not independently associated with blood pressure or risk of hypertension among 1,420 Chinese participants. The inconsistency among these Chinese studies may be partially due to different study populations, geographic, and seasonal differences. In our study, serum 25(OH)D was significantly lower than those in three other studies. Further more, some randomized clinical trials of vitamin D supplementation that did not improve BP or reduce hypertension. Witham *et al*. [33]suggested that 6 months of intermittent, high-dose oral vitamin D3 did not reduce blood pressure or left ventricular mass in patients with resistant hypertension. In postmenopausal women, vitamin D_3_ supplementation did not reduce either blood pressure or the risk of developing hypertension over 7-years of follow-up[34]. For our meta-analysis, the included population in these studies most is foreigners even white. More meta-analysis on China's population is needed.

Although we did not find any associations of serum 25(OH)D levels with blood pressure or risk of incident hypertension, previous experimental and population studies have provided several mechanisms to explain the relationships between serum 25(OH)D and blood pressure. The mechanism of this effect may involve ability of vitamin D to negatively regulate the renin-angiotensin system (RAS) and the association with endothelial vasodilator dysfunction[8,9,35–39]. In vitamin D deficient animals there is an increased activation of the RAS system was observed in vitamin D-deficient animals and has been linked to increased incidence of hypertension, left ventricular hypertrophy, and atherosclerosis[9,36,37,40]. Moreover, vitamin D could affect lipid metabolism indirectly through an effect on insulin secretion and sensitivity[41,42].

Strengths of our study are the use of a large, community-based cohort without pre-existing prevalent hypertension at baseline and a considerable length of follow-up. These findings add new knowledge to underlying hypertension mechanisms and strengthen the case for disturbances in low 25(OH)D as a cardiovascular risk factor. Numerous studies and meta-analyses have suggested that vitamin D deficiency has a negative association with hypertension; however, this effect has few been studied in Asians, especially the Chinese.

### Limitation

The present study has some significant limitations: First, 1,25-Dihydroxyvitamin D3 is the active metabolite. But 1,25-Dihydroxyvitamin D3 was not measured in present study. Second, PTH has been linked to hypertension in previous studies, independent of vitamin D levels[43]. Unfortunately, we did not have PTH levels available in our study to assess the independent role of PTH in the remaining ethnic differences in blood pressure. Third, the gold standard is now to use LC-Mass Spec to measure 25(OH)D but we use the ELISA. Moreover, nutritional intake may also influence the risk of hypertension. Although we had information on salt habit and alcohol intake, our data on nutritional diet are limited.

## Conclusion

In conclusion, our study suggests that serum 25-hydroxyvitamin D levels are not independently associated with blood pressure or risk of incident hypertension in a Chinese population in prospective study. Prospective population studies are needed to evaluate the role of serum 25(OH)D in the incidence of hypertension. PRISMA 2009 Checklist preferred reporting Items for systematic reviews and Meta-analyses(S1 Table).

## Supporting information

S1 Table PRISMA 2009 checklist. Preferred reporting items for systematic reviews and meta-analyses.
(DOC)
